# Cordycepin Inhibits Growth and Metastasis Formation of MDA-MB-231 Xenografts in Nude Mice by Modulating the Hedgehog Pathway

**DOI:** 10.3390/ijms231810362

**Published:** 2022-09-08

**Authors:** Wenya Wu, Xiaomin Li, Meng Qi, Xin Hu, Fenghua Cao, Xiaoping Wu, Junsheng Fu

**Affiliations:** 1 College of Life Sciences, Fujian Agriculture and Forestry University, Fuzhou 350002, China; 2 Mycological Research Center, Fujian Agriculture and Forestry University, Fuzhou 350002, China

**Keywords:** *Cordyceps militaris*, cordycepin, triple-negative breast cancer, Hedgehog pathway

## Abstract

We previously found that cordycepin inhibits the growth and metastasis formation of MDA-MB-231 cells through the Hedgehog pathway but has not validated this in vivo. In this study, we confirmed cordycepin’s anti-triple-negative breast cancer (TNBC) effect in nude mice and documented its mechanism. We found that cordycepin reduced the volume and weight of MDA-MB-231 xenografts and affected the expression of proliferation-, apoptosis-, epithelial–mesenchymal transition-, and matrix metalloproteinase-related proteins without side effects. RNA sequencing screening, pathway enrichment, and the protein network interaction analysis revealed enriched pathways and targets mainly concentrated on the Hedgehog pathway and its core components of SHH and GLI2. This indicates that the Hedgehog pathway plays a central role in the cordycepin-mediated regulation of growth and metastasis formation in TNBC. The database analysis of the Hedgehog pathway markers (SHH, PTCH1, SMO, GLI1, and GLI2) revealed that the Hedgehog pathway is activated in breast cancer tissues, and its high expression is not conducive to a patient’s survival. Finally, we verified that cordycepin effectively inhibited the Hedgehog pathway in TNBC through Western blotting and immunohistochemistry. This study found that cordycepin could regulate the growth and metastasis formation of TNBC through the Hedgehog pathway in vivo, which provides new insights for targeting and treating breast cancer.

## 1. Introduction

*Cordyceps militaris* is a nutrient-rich edible fungus containing cordycepin; cordyceps polysaccharide; pentostatin; ergosterol; and other active components with pharmacological effects, such as antibacterial, antiviral, antitumor, immunity regulation, lipid-lowering, antiplatelet agglutination, and memory improvement activities [1,2,3,4]. Due to its various physiological activities, it is widely used as a natural medicine and folk tonic food at home and abroad [5]. Its active ingredients, nutritional value, and medicinal functions are very similar to those of the wild *Cordyceps sinensis*, but its factory production and relatively low price make it the best substitute [6].

One of the earliest and arguably most important natural products isolated from *Cordyceps militaris* is the adenosine analog cordycepin (3-deoxyadenosine) [7]. *Cordyceps militaris* contains more cordycepin than *Cordyceps sinensis*. Most of the cordycepin used in the current study was extracted from a *Cordyceps militaris* fermentation broth. Cordycepin has antitumor, antivirus, and immune regulation effects (among others) and is used to treat leukemia [8]. Cordycepin induces apoptosis and inhibits the growth of various tumors, which has made it an antitumor research hotspot [9]. Although many studies have reported the anticancer effects of cordycepin, none have documented them on a molecular biology or even signaling pathways level.

Breast cancer is one of the most common malignant tumors in women worldwide, ranking among the top three gynecological tumors in the world in terms of both morbidity and mortality. Furthermore, triple-negative breast cancer (TNBC) is more prone to metastasis and difficult to treat than other breast cancers due to the absence of an estrogen receptor, progesterone receptor, and human epidermal growth factor receptor 2 on its surface [10]. Currently, triple-negative breast cancer treatment is ineffective and has more side effects. Therefore, understanding the molecular mechanisms allowing breast cancer proliferation inhibition and developing new drugs for TNBC is crucial.

Our group previously demonstrated, through in vitro experiments, that cordycepin induced apoptosis and regulated the apoptotic factors in breast cancer cells; affected the expression of motility-, migration, invasion-, and epithelial–mesenchymal transition (EMT)-related proteins; inhibited the expression of the Hedgehog pathway components and GLI transcription activity; and knocking out GLI blocked the effects of cordycepin on apoptosis, EMT, and the Notch pathways, suggesting that the Hedgehog pathway and its related targets play an important role in breast cancer inhibition [11]. Thus, we conducted this study to further document the antitumor effect of cordycepin, clarify the inhibitory effect of cordycepin on breast cancer, and explore the role of the Hedgehog and other signaling pathways. We investigated the inhibitory effect and mechanism of cordycepin on TNBC by constructing xenograft tumors in nude mice and analyzing the protein expression levels and cancer tissue. We hope to accumulate experimental evidence for the study of the occurrence and development mechanism and the clinical treatment of TNBC.

## 2. Results

### 2.1. Cordycepin Inhibits the Growth of MDA-MB-231 Xenografts in Nude Mice

**Figure 1:**
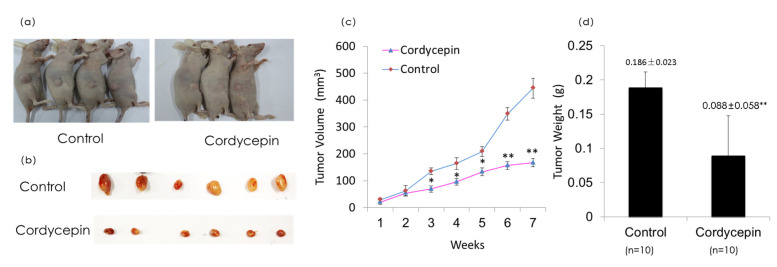
Effect of cordycepin on the growth of xenografts in nude mice. (**a**) The picture of BALB/C nude mice with tumors. Tumors in nude mice injected with MDA-MB-231 cells in vivo, administered with cordycepin (ig) and sterile water (ig) for 7 weeks. (**b**) The appearance of the MDA-MB-231 tumor detached from a nude mouse. The weight of the cordycepin group was more significantly reduced than that of the control group. (**c**) Effects of cordycepin on the tumor volumes of mice. Within 7 weeks of administration, the length and width of the tumors were measured once a week, and the tumor volume was calculated by (D × d^2^)/2. The data represent the mean  ±  SD. (**d**) Tumor weights. The data represent the mean  ±  SD. *n* = 10. Compared with the control group. * *p* < 0.05 and ** *p* < 0.01.

We first measured the effects of cordycepin on the growth of MDA-MB-231 xenografts. This study used 20 nude mice to establish a human breast cancer MDA-MB-231 xenograft model, and 20 of them developed tumors (the tumor formation rate was 100%). The skin surface of the mice at the tumor formation site was pink, and the xenograft was raised on the skin surface with an irregular shape, clear boundary, and rough texture (Figure 1a,b). Cordycepin inhibited MDA-MB-231 tumor growth (volume and weight) in nude mice (Figure 1c,d). The control and cordycepin groups had significantly different xenograft volumes at week 3. At week 7, the control group xenografts were 3.5 times larger and 2.6 times heavier than those of the cordycepin group. Noteworthily, the mice had normal diets, behaviors, and normal mental states during the administration period. Cordycepin did not affect the body weights of the MDA-MB-231 tumor-bearing mice (Table 1). These results suggest that cordycepin is an effective drug for the treatment of TNBC, and it is safe and nontoxic.

### 2.2. Cordycepin Inhibits Proliferation and Induces Apoptosis in MDA-MB-231 Xenografts

**Figure 2:**
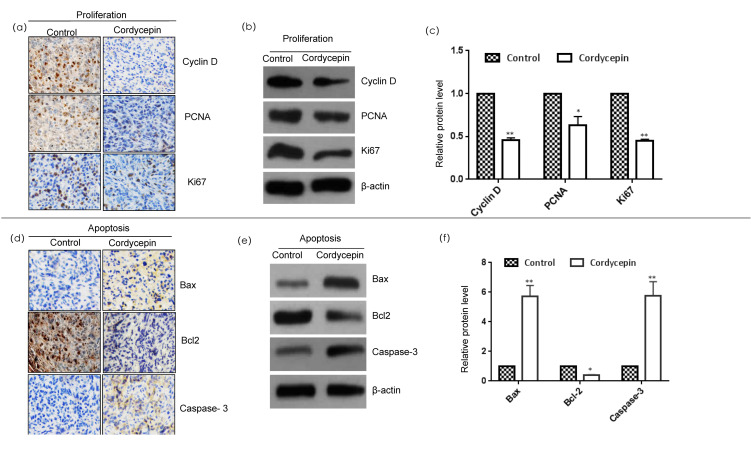
Effect of cordycepin on the proliferation and apoptosis of MDA-MB-231 xenografts. (**a**) Immunohistochemical expression of proliferation-related proteins. Tumor sections were subjected to IHC using cyclin D, PCNA, and Ki67 antibodies. (**b**) Western blot analysis of proliferation-related proteins. The levels of cyclin D, PCNA, and Ki67 in MDA-MB-231 xenografts fed 7 weeks with cordycepin and sterile water, respectively, were assessed by a Western blot analysis; (**c**) relative protein levels of cyclin D, PCNA, and Ki67. The relative protein level in each condition in (**b**) was quantitated using ImageJ. Data are expressed as the mean  ±  SD. (**d**) Immunohistochemical expression of apoptosis-related proteins. Tumor sections were subjected to IHC using Bax, Bcl-2, and caspase-3 antibodies. (**e**) Western blot analysis of proliferation-related proteins. The levels of Bax, BCL2, caspase-3 in MDA-MB-231 xenografts fed 7 weeks with cordycepin and sterile water, respectively, were assessed by a Western blot analysis; (**f**) relative protein levels of Bax, BCL2, caspase-3. The relative protein level in each condition in (**e**) was quantitated using ImageJ. Data are expressed as the mean  ±  SD. * *p* < 0.05 and ** *p* < 0.01.

The occurrence and development of tumors are closely related to the activation of oncogenes, inactivation of tumor suppressor genes, and abnormal expression of apoptosis genes and cell proliferation genes; breast cancer is no exception [12]. To confirm that cordycepin can effectively affect the proliferation and apoptosis of breast cancer, we first measured the effect of cordycepin on the expression of proliferation-related proteins (cyclin D, PCNA, and Ki67) and apoptosis-related proteins (Bcl-2, Bax, and caspase-3) in tumor tissues by immunohistochemistry and Western blot analysis (Figure 2). In recent years, PCNA, Ki-67, and cyclin D have been widely used as cell proliferation markers. Cordycepin inhibits the expression of these three markers in breast cancer tissue and inhibits breast cancer proliferation. The Bcl-2 family is the most common apoptosis regulation family, and its members mainly include Bcl-2, Bax, and caspase-3. Cordycepin can activate the expression of the proapoptotic factor Bax and the apoptosis promoter and executor caspase-3 and inhibit the expression of the antiapoptotic factor Bcl-2. These results show that cordycepin inhibited the proliferation and induced the apoptosis of breast cancer cells by affecting the proliferation markers (PCNA, Ki-67, and cyclin D) and apoptosis factors (Bcl-2, Bax, and caspase-3).

### 2.3. Cordycepin Inhibits Invasion and Metastasis of MDA-MB-231 Xenograft

**Figure 3:**
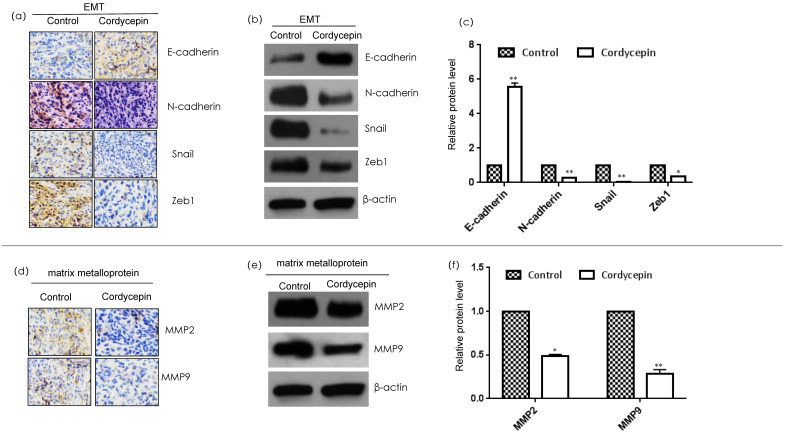
Effect of cordycepin on EMT markers and MMPs in MDA-MB-231 xenografts. (**a**) Immunohistochemical expression of proliferation-related proteins. Tumor sections were subjected to IHC using E-cadherin, N-cadherin, Snail, and Zeb1 antibodies. (**b**) Western blot analysis of proliferation-related proteins. The levels of E-cadherin, N-cadherin, Snail, and Zeb1 in MDA-MB-231 xenografts fed 7 weeks with cordycepin and sterile water, respectively, were assessed by a Western blot analysis; (**c**) relative protein levels of E-cadherin, N-cadherin, Snail, and Zeb1. The relative protein level in each condition in (**b**) was quantitated using ImageJ. Data are expressed as the mean  ±  SD. (**d**) Immunohistochemical expression of apoptosis-related proteins. Tumor sections were subjected to IHC using MMP2 and MMP9 antibodies. (**e**) Western blot analysis of proliferation-related proteins. The levels of MMP2 and MMP9 in MDA-MB-231 xenografts fed 7 weeks with cordycepin and sterile water, respectively, were assessed by a Western blot analysis; (**f**) relative protein levels of MMP2 and MMP9. The relative protein level in each condition in (**e**) was quantitated using ImageJ, Data are expressed as the mean  ±  SD. * *p* < 0.05 and ** *p* < 0.01.

Matrix metalloproteins and EMT are important factors of tumor cell metastasis and invasion [13]. Therefore, we assessed the effect of cordycepin on the expression of EMT-related proteins (N-cadherin, E-cadherin, snail, and ZEB1) and matrix metalloproteinases (MMP2 and MMP9) in tumor tissues by immunohistochemistry and Western blotting (Figure 3). Cordycepin inhibited the expression of N-cadherin, snail, and ZEB1 in breast cancer and enhanced the expression of E-cadherin. Meanwhile, cordycepin inhibited the expression of MMP2 and MMP9. These results indicate that cordycepin can inhibit breast cancer invasion and metastasis by affecting MMPs (MMP2 and MMP9) and EMT-related proteins (N-cadherin, E-cadherin, snail, and ZEB1).

### 2.4. Cordycepin Inhibition of Breast Cancer Is Related to the Hedgehog Pathway

**Figure 4:**
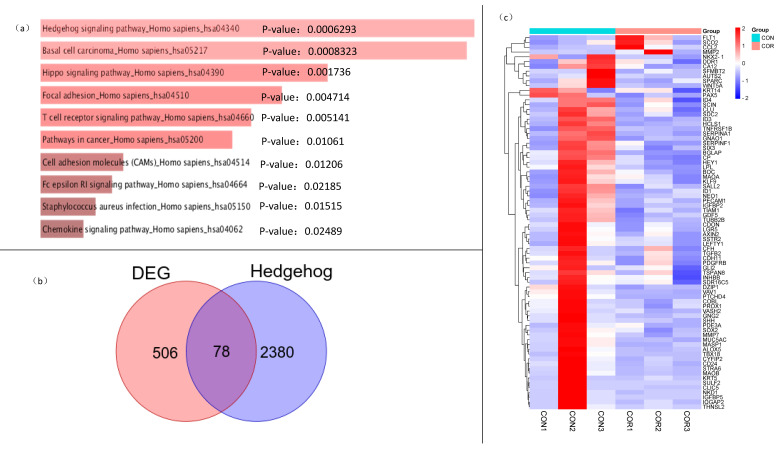
Analysis of the differentially expressed genes, pathway enrichment, and intersection with Hedgehog pathway genes. (**a**) Enrichment of the growth and metastasis-related gene pathways. Growth- and metastasis-related terms in the 584 genes were obtained by the Enrichr online enrichment analysis to obtain the relevant pathways and ranked according to the *p*-values. (**b**) Intersection of the differentially expressed genes and Hedgehog pathway genes. Seventy-eight represents the number of intersecting genes, and the orange- and purple-colored circles represent five hundred and eighty-four differentially expressed genes and two thousand, four hundred and fifty-eight Hedgehog pathway genes, respectively. (**c**) Heatmaps of 78 intersecting genes expressions. A hierarchical clustering tree indicates the gene expression pattern similarities of the 78 genes between the experimental and control groups. The expression levels of the 270 genes are shown by different color lumps: red, high (upregulated); white, medium; and blue, low (downregulated).

To further document the mechanism of cordycepin-induced growth and metastasis formation inhibition in breast cancer, we screened two groups of differentially expressed genes by sequencing the RNA of the xenografts from the control and administration groups. We screened 584 genes (screening parameters: *p* < 0.05 and log2 (FC) < 1) and then performed the KEGG and GO function analyses. In line with the fast growth and easy metastasis formation of tumors, we found terms related to growth and metastasis among the significantly enriched GO. We analyzed the growth and metastasis differential genes by an Enrichr online enrichment analysis, and the results showed (Figure 4a) that the most enriched signaling pathway for growth and metastasis-related genes was the Hedgehog pathway, followed by others such as basic cell carcinoma, the Hippo signaling pathway, and focal adhesion. Aberrant activation of the Hedgehog pathway, including through genetic mutations [14,15], can lead to excessive cell proliferation, promoting the development of various tumors. Numerous studies have confirmed that the abnormal activation of the Hedgehog pathway is not only closely related to tumor cell the EMT, Notch, and Hippo signaling pathways [16,17,18] but also participates in the occurrence, growth, differentiation, and proliferation of TNBC. Seventy-eight intersecting genes (Table S1) were obtained by intersecting five hundred and eighty-four genes with genes in the Hedgehog pathway in the database (Figure 4b). There were significant differences in the expression of these genes in the MDA-MB-231 xenograft in nude mice (*p* < 0.05) (Figure 4c). Therefore, cordycepin most likely regulates breast cancer growth through the Hedgehog pathway.

### 2.5. Analysis of Key Genes in the Protein–Protein Interaction Network

**Figure 5:**
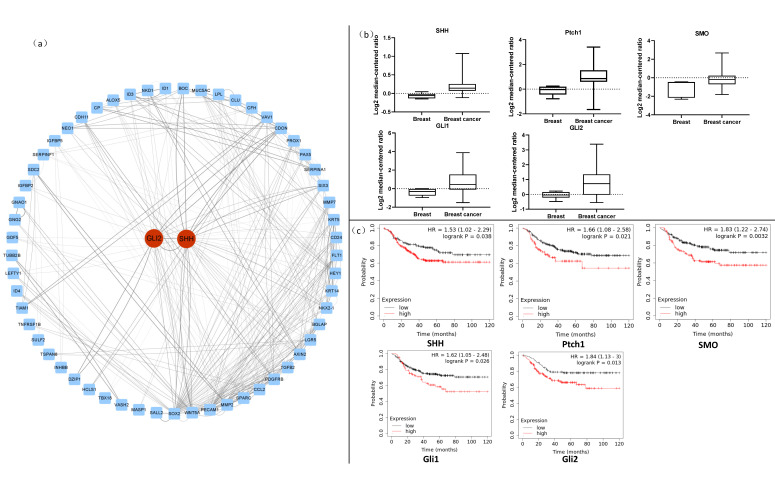
Protein–protein interaction analysis of intersecting targets, and a database analysis of key targets in the Hedgehog pathway. (**a**) Protein–protein interaction network diagram. The PPI data were downloaded from the Spring database and visualized using Cytoscape; the red nodes are the core targets of the Hedgehog pathway, and the blue nodes are other intersection targets. (**b**) Oncomine database analysis. Box plots derived from gene expression data in Oncomine comparing the expression of SHH, Gli1, Gli2, Ptch1, and SMO genes in normal (left plot) and breast cancer tissues (right plot). (**c**) Kaplan–Meier analysis. Kaplan–Meier plots showing the overall survival in breast cancer. In red: patients with expression above the median; in black: patients with expressions below the median.

We drew the protein–protein interaction network map of 78 genes and found that the core genes SHH and GLI2 of the Hedgehog signaling pathway had high degree values (Figure 5a). High degree values indicate important genes playing a key role. The Hedgehog pathway is mainly composed of Hedgehog ligands (SHH), Patched trans-membrane proteins (PTCH1), Smoothened (SMO), and intracellular transcription factors GLI1 and GLI2. PTCH1 and SMO are located on cell membranes, and the former are Hedgehog receptors that bind to various Hedgehog ligands. In the absence of Hedgehog ligands, PTCH1 are not restricted and inhibit SMO activity, and Hedgehog signals cannot enter the nucleus to activate the transcription of downstream target genes. Meanwhile, when the Hedgehog ligand is excessively secreted, it can bind to PTCH1 and prevent SMO inhibition, leading GLI to enter the nucleus and activate the expression of downstream target genes [19,20]. Thus, the Hedgehog signaling pathway and its core members, such as SHH and GLI2, play an essential role in the growth regulation of breast cancer.

### 2.6. Breast Cancer Tissue and Normal Breast Tissue Differently Express Hedgehog Pathway Markers

To clarify the clinical significance of the Hedgehog pathway in the treatment of TNBC, we used the Oncomine database and Kaplan–Meier analysis and assessed the expression of key factors (SHH, PTCH1, SMO, GLI1, and GLI2) of the Hedgehog pathway in normal breast tissue and breast cancer tissue, along with their relationship to the prognosis of breast cancer patients. First, we analyzed the expression of SHH, PTCH1, SMO, GLI1, and GLI2 in normal breast and breast cancer tissues through the Oncomine database. We found that normal breast tissue and breast cancer tissue expressed significantly different levels of SHH, PTCH1, SMO, GLI1, and GLI2 (*p* < 0.05, Figure 5b). Their expression levels were all higher in breast cancer tissue than in normal breast tissue, with more than a two-fold difference for SMO, GLI1, and GLI2. Thus, breast cancer tissues express high levels of the Hedgehog pathway factors.

### 2.7. Hedgehog Pathway Markers Are Associated with Prognosis in Breast Cancer Patients

In clinical practice, TNBC is characterized by rapid disease progression, strong invasiveness, and high recurrence and metastasis rates. Therefore, we used the Kaplan–Meier plotter website to analyze the effects of SHH, PTCH1, SMO, GLI1, and GLI2 on the prognosis of breast cancer patients. In addition to PTCH1, high SHH, SMO, GLI1, and GLI2 expression levels were all associated with a poor prognosis in breast cancer patients (Figure 5c). Therefore, we speculate that the Hedgehog pathway plays a critical role in the invasion and metastasis formation of breast cancer.

### 2.8. Cordycepin Inhibits Hedgehog Pathway Markers in Triple-Negative Breast Cancer

**Figure 6:**
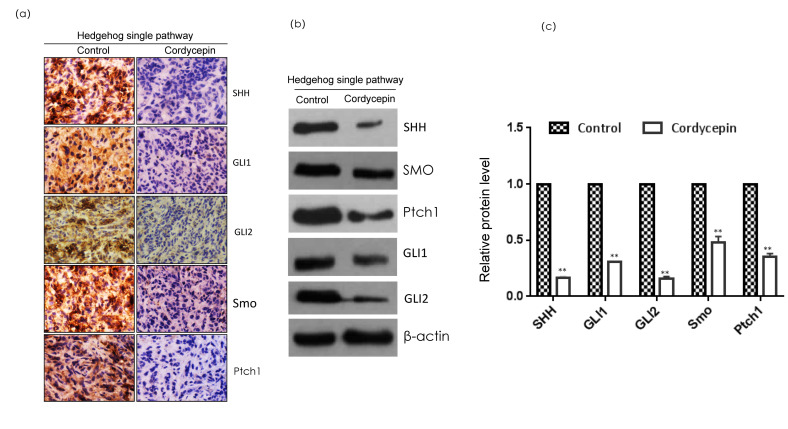
Expression of the Hedgehog pathway markers in a MDA-MB-231 xenograft. (**a**) The immunohistochemical expression results. The tumor sections were subjected to IHC using antibodies. (**b**,**c**) The expression results of the Western blot. The levels of SHH, Gli1, Gli2, Ptch1, and SMO in MDA-MB-231 xenografts fed 7 weeks with cordycepin and sterile water, respectively, were assessed by a Western blot analysis; the relative protein level (**c**) in each condition in (**b**) was quantitated using ImageJ. Data are expressed as the mean  ±  SD. ** *p* < 0.01.

Through RNA sequencing and database analysis, we found that breast cancer tissues express abnormally high levels of the Hedgehog pathway members, and the activation of the Hedgehog pathway is also closely related to a poor prognosis for breast cancer patients. This indicates that the Hedgehog pathway plays an important role in the occurrence and development of breast cancer. Therefore, we confirmed that cordycepin inhibited growth and metastasis formation in breast cancer by regulating the Hedgehog pathway through immunohistochemistry and Western blotting (Figure 6). Moreover, cordycepin significantly inhibited the expression of SHH, PTCH1, SMO, GLI1, and GLI2, which are central components of the Hedgehog pathway, suggesting that cordycepin can inhibit the Hedgehog pathway and TNBC.

## 3. Discussion

In recent years, research on the antitumor effect of cordycepin has gradually gained traction and revealed inhibitory effects on lung, liver, breast, gastric, and other types of cancer [21]. Some studies have found that cordycepin significantly inhibited breast cancer growth. Dong et al. [22] found that cordycepin promoted G2/M arrest and apoptosis in MCF-7 and MDA-MB-231 cells, thereby inhibiting the proliferation of irradiated cells in vitro and in vivo. Lee et al. [23] and Wang et al. [24] treated MCF-7 and MDA-MB-231 cells with cordycepin, conducted in vitro culture and xenotransplantation experiments in nude mice, and found that cordycepin induced tumor cell apoptosis, thereby inhibiting breast cancer development. At the same time, studies have found that cordycepin can inhibit the EMT pathway in triple-negative breast cancer, thereby inhibiting breast cancer metastasis, and has a significant effect on the treatment of triple-negative breast cancer [25]. Our team previously showed that cordycepin inhibited growth, apoptosis, motility, migration, invasion, and EMT markers expression in human breast cancer cells (MDA-MB-231) in vitro. In addition, cordycepin inhibited GLI transcription and expression of the Hedgehog pathway components in breast cancer cells [11]. In this study, we investigated the inhibitory effect of cordycepin on breast cancer by constructing mouse breast cancer xenografts. In the cordycepin group, the xenografts grew more slowly and yielded smaller and lighter tumors than in the control group. All the results indicated that cordycepin effectively inhibited the growth of mouse breast cancer xenografts, which is consistent with our previous experimental results [11]. In conclusion, cordycepin effectively inhibited breast cancer cell proliferation in vivo and in vitro.

Next, we measured the expression levels of tumor-related proteins. PCNA, Ki-67, and cyclin D are nuclear antigens related to cell proliferation and have been widely used as cell proliferation markers in recent years [26,27,28]. The Bcl-2 family is the most common apoptosis-regulating family, and its members mainly include Bcl-2 and Bax. The antiapoptotic protein Bcl-2 can directly bind to Bax, inactivating it and inhibiting the caspase-3 apoptotic activity, thus impeding apoptosis. Some malignant tumor cells escape apoptosis by increasing the expression of antiapoptotic proteins of the Bcl-2 family, enhancing proliferation [29]. EMT can enable tumor cells to acquire high mesenchymal phenotypes such as proliferation, migration, invasion, antiapoptosis, and extracellular matrix degradation; it is a pivotal biological process for malignant tumor cells to acquire migration and invasion ability [30]. MMP-2 and MMP-9 are most closely related to tumor invasion and metastasis [31]. The cordycepin group in our study had higher tumor-related protein (cyclin D, PCNA, and Ki-67) expression levels than the control group. It also had lower apoptosis-related protein Bcl-2 expression levels and higher Bax and caspase-3 expression levels. Regarding EMT-related proteins, N-cadherin, snail, and Zeb1 were upregulated in the cordycepin group, while E-cadherin was downregulated. Finally, the cordycepin group had lower matrix metalloprotein-related protein levels (MMP2 and MMP9). These results indicate that cordycepin could inhibit breast cancer by regulating the expression of proliferation-, apoptosis-, EMT-, and matrix metalloprotein-related targets.

After confirming that cordycepin inhibited breast cancer, we screened the differentially expressed genes obtained by the RNA sequence analysis and performed a composition analysis and pathway enrichment of the screened differential genes. We found that, among the genes related to growth and metastasis, the Hedgehog pathway was the most enriched pathway. Combined with the previous reports indicating that cordycepin inhibits breast cancer in vitro, this relation with the Hedgehog pathway and its core target GLI1 suggests that the Hedgehog pathway plays an important role in the cordycepin-induced inhibition of breast cancer growth and metastasis formation. After collecting the related genes of the hedgehog pathway in the database and intersecting the differential genes, we identified 78 intersecting proteins. The protein interaction network revealed that the two core genes of the Hedgehog pathway, SHH and GLI2, had a high degree of intersection and were important targets. This result confirmed that the Hedgehog pathway is closely related to the regulation of TNBC by cordycepin.

The Oncomine database and Kaplan–Meier Plotter website are clinical tumor databases. Since cordycepin inhibited breast cancer growth in vitro, we aimed to confirm the relationship between the Hedgehog pathway and the inhibition of breast cancer growth using these clinical tumor databases. The Oncomine database integrates RNA and DNA-seq data from TCGA (The Cancer Genome Atlas), GEO (Gene Expression Omnibus), and the published literature; it is a gene-on-a-chip database that analyzes their differential expression in cancer and normal tissues [32,33]. The Kaplan–Meier Plotter website is the world’s most widely accepted online prognostic analysis database [34]. Using these databases, we analyzed the expression of the Hedgehog pathway in normal and breast cancer tissues and the relationship between the prognostic and Hedgehog pathways in breast cancer patients. The results showed that the Hedgehog pathway was activated in tumor tissues, and the expression levels of the key genes were higher than those in normal tissues. Moreover, the expression level of the Hedgehog pathway is correlated with the survival rate of patients with TNBC. When the Hedgehog pathway is highly expressed, the survival rate of patients is significantly reduced, and continuous tumor growth and metastasis formation are some of the important factors leading to the death of tumor patients. In addition, according to literature reviews, GLI1, the Hedgehog pathway effector, directly regulates the expression of EMT transcription factors in TNBC cells, and the activation of Hedgehog signaling can enhance the proliferation, invasion, and migration ability of TNBC. Contrarily, inhibiting the expression of the Hedgehog pathway reduces the cloning, self-renewal, and movement ability of TNBC [35]. Finally, we verified that cordycepin inhibited the Hedgehog pathway at the protein level by immunohistochemistry and Western blotting. In conclusion, cordycepin can regulate MDA-MB-231 cell growth and metastasis formation by blocking the Hedgehog pathway.

## 4. Materials and Methods

### 4.1. Chemicals and Materials

We purchased MDA-MB-231 cells from iCell Bioscience Inc. (Shanghai, China). We obtained 20 BALB/C nude mice (SPF grade, all female, weight 10–15 g, age 3–5 weeks), feed, litter, and cages from Wu’s laboratory animals (Fuzhou, China) (www.wssydw.com, accessed on 17 March 2017). We bought doxorubicin from Sigma (San Francisco, CA, USA). We purchased RPMI 1640 medium and fetal bovine serum from Gibco (Grand Island, NY, USA). We obtained NaCl, KCl, KH_2_SO_4_, absolute ethanol, xylene, H_2_O_2_, and neutral gum from Sinopharm Chemical Reagent Co., Ltd (Shanghai, China). We purchased Trypsin K from Roche (Basel, CH). We obtained phosphate-buffered solution (PBS); hematoxylin dye; EDTA (pH 9.0) antigen retrieval solution; bovine serum albumin (BSA); hematoxylin differentiation solution; hematoxylin blue liquid; neutral gum; citrate buffer; primary antibodies (against ki-67, proliferating cell nuclear antigen (PCNA), caspase-3, E-cadherin, N-cadherin, snail, ZEBI, mmp2, mmp9, cyclin D, and Bax); secondary antibody (HRP-conjugated goat anti-rabbit IgG (H+L)); and histochemistry kit DAB chromogenic reagent from servicebio(Wuhan, China) (www.servicebio.cn, accessed on 11 April 2017). We purchased Trizol from Life Technologies. Finally, we bought chloroform, phenol, isopropanol, ethanol, and agarose from Guangzhou Chemical Reagent Factory (Guangzhou, China) (www.chemicalreagent.com, accessed on 20 January 2017)).

### 4.2. Effect of Cordycepin on the Growth of MDA-MB-231 Cell Xenografts

We diluted the MDA-MB-231 cells to 1 × 10^8^ cells/mL with a serum-free cell culture medium and mixed them with BD Matrigel (1:1) on ice for later use. We reared the nude mice in a sterile environment for one week, then injected the mixture of cells and BD Matrigel subcutaneously using a 1-mL syringe. We monitored the tumor formation daily. When the tumors on the tumor-bearing area on the back of the mice reached the size of a mung bean, we randomly divided the 20 mice into a control group and an administration group. Based on our previous in vitro experiments and data from the literature, the control group received sterile water in the stomach daily, and the administration group received 20 mg/kg of cordycepin daily.

We observed the growth of the nude mice daily, recorded their weight weekly, and noted any mental sluggishness signs and weight loss. After administration, we checked the tumor volume with Vernier calipers every other week (tumor volume = (D × d^2^)/2, where D is the long diameter of the tumor, and d is the short diameter of the tumor). We used the average tumor volume of each group to draw a tumor growth curve. Seven weeks after the administration, we photographed the mice, sacrificed them, collected the tumor tissue, and weighed the xenografts.

### 4.3. RNA Isolation

RNA was extracted from liver tissue using the conventional method of Trizol [36]. RNA concentration and purity were determined using the NanoDrop2000 spectrophotometer (Thermo Fisher Scientific, (Waltham, MA, USA)). The integrity of the nucleic acid samples was tested by 1% agarose gel electrophoresis. An appropriate amount of liver tissue is thoroughly ground in liquid nitrogen and transferred to a 1.5-mL centrifuge tube, then 1 mL Trizol is added and mixed. After adding 200 μL chloroform, samples were centrifuged for 10 min, 12,000× *g* at 4 °C, and the upper aqueous phase was taken. After adding an equal volume of phenol:chloroform (25:24), the samples were centrifuged for 10 min, 12,000× *g* at 4 °C, and the upper aqueous phase was taken. Samples were added to an equal volume of chloroform, mixed well, and centrifuged for 10 min, 12,000× *g* at 4 °C. RNA was precipitated by an equal volume of Isopropanol and kept for 1 h at −20 °C, then centrifuged for 10 min, 12,000× *g* at 4 °C. Precipitation was washed by 1 mL 75% ethanol and centrifuged for 5 min, 800× *g* at 4 °C, and the upper aqueous phase was taken. Ethanol was sucked up and vacuum-dried for 2 to 3 min. Add 20–50 μL RNAse-free water to the sample, put it in a bath at room temperature for 10 min, then centrifuge instantly. RNA samples were stored at −80 °C.

### 4.4. Western Blotting

We prepared lysates from the xenografts using a homogenizer and lysis solution. We obtained a protein standard curve using BSA and Coomassie brilliant blue solution in a microplate reader. We diluted the lysate to reach the concentration range of the standard curve and then determined its concentration. We prepared a separation gel with a lower layer containing 10% ammonium persulfate and 10 mL of 10% polyacrylamide and an upper layer of concentrated glue containing 6 mL of 5% polyacrylamide and filled with neutral balsam. After the gel solidified, we added the samples, then placed it in the electrophoresis tank and added the electrophoresis buffer to start running the gel.

After transferring the membrane, we took it out and placed it in a petri dish. We washed the membranes three times with tris-buffered saline with Tween 20 (TBST). Next, we prepared 5% skimmed milk powder with TBST to seal the membrane; added the diluted primary antibody (against ki-67, PCNA, caspase-3, E-cadherin, N-cadherin, snail, ZEBI, MMP2, MMP9, cyclin D, and Bax); and incubated it overnight at 4 °C. We then washed the membranes three times with TBST and then incubated them with the secondary antibody (HRP-conjugated goat anti-rabbit IgG (H+L)) for 2 h. Finally, we used a BCIP/NBT color development kit in a dark place for about 10 min to reveal the color. We recorded the results with a gel imaging system.

### 4.5. Immunohistochemistry

We fixed liver tissue specimens in formalin and embedded them in paraffin. We incubated the sections for 60 min at room temperature, added xylene, incubated 10 min, and then added xylene again and incubated 10 min to melt the wax. Then, we rehydrated the slides by immersing them in ethanol absolute, 95% ethanol, 85% ethanol, 75% ethanol, 50% ethanol, and pure water for 5 min each. After washing three times with PBS, we added 3% H_2_O_2_, incubated in a wet box for 10 min, and then washed off the H_2_O_2_ on the slices with PBS. After that, we immersed the slides in citrate buffer and placed them in a stainless-steel pot for 20 min to achieve antigen retrieval. We then added 10% goat serum dropwise and sealed the slides in a humidified box at room temperature for 30–60 min. Then, we incubated the tissue sections with the primary antibodies overnight at 4 °C. After washing the sections twice with PBS to remove the primary antibodies, we added the secondary antibody (biotin-labeled goat anti-mouse/rabbit) and incubated it at 37 °C for 30 min. We next dyed and stained the sections with SABC, DAB, and hematoxylin. After staining, we put the slices in 1% hydrochloric acid alcohol for a few seconds, then in 50% ethanol, 75% ethanol, 85% ethanol, 95% ethanol, and absolute ethanol for 5 min each. Finally, we mounted the sections using neutral balsam, inspected them, and took photographs.

### 4.6. Oncomine Database Analysis

We used the Oncomine database to analyze the mRNA expression levels of growth and metastasis differential genes screened in the early stage in the clinical database. The Oncomine (www.oncomine.org, (accessed on 15 October 2020) (Ann Arbor, MI, USA)) database can collect and analyze microarray data of gene expression profiles in tumor samples, providing real-time transcriptome data. Currently, Oncomine includes 65 gene expression datasets, more than 4700 microarrays, and 48,000,000 gene expression results, primarily for the differential expression analysis of most cancer types, the corresponding normal tissues, and various cancer subtypes. It can be used to analyze the differential expression analyses of multiple studies and draw relatively comprehensive conclusions. Inputting gene names, setting fold changes, and sequencing of the *p*-value can yield differential expression data of this gene in various tumors. Thus, selecting breast cancer can yield the expression levels of target genes in tumor tissues and normal tissues.

### 4.7. Kaplan–Meier Analysis

We logged in to the kmplot website (http://kmplot.com/analysis/, accessed on 15 October 2020 (Budapest, HU)), clicked on the breast cancer related database, and entered the target gene. Next, we divided these cohorts into two groups according to their median gene expression levels, and assessed the relationship between the genes expression levels and the survival of breast cancer patients. We performed this for each of the genes that interested us. 

### 4.8. Data Analysis

We processed data with the SPSS 23.0 statistical software package and compared the groups by one-way ANOVA, with *p* < 0.05 indicating statistically significant differences. We quantitatively analyzed the Western blot results using ImageJ2X (Bethesda, MD, USA).

## 5. Conclusions

We established a nude mouse xenograft model to confirm that cordycepin significantly inhibited the expression level of the factors related to growth, apoptosis, metastasis formation, and other processes in TNBC. An analysis of the RNA sequencing data revealed that the Hedgehog pathway was the most enriched pathway in breast cancer tissues. By analyzing the markers of the Hedgehog pathway and assessing their expression changes in xenografts, we confirmed that the Hedgehog signaling pathway plays an important role in the anti-breast cancer effect of cordycepin. In summary, cordycepin can regulate growth and metastasis formation in TNBC by inhibiting the Hedgehog pathway.

## Figures and Tables

**Table 1:** Effects of cordycepin on body weights of nude mice with MDA-MB-231 xenografts.

Time	Control (g)	Cordycepin (g)
Week 1	19.42 ± 1.37	21.23 ± 0.58
Week 2	20.02 ± 1.32	21.16 ± 0.71
Week 3	20.46 ± 1.35	20.51 ± 0.72
Week 4	21.17 ± 0.56	21.17 ± 0.48
Week 5	21.27 ± 1.08	21.08 ± 0.56
Week 6	21.33 ± 1.22	21.53 ± 0.68
Week 7	21.43 ± 0.78	21.31 ± 0.87

## Data Availability

The data that support the findings of this study are available from the corresponding author, Junsheng Fu, upon reasonable request.

## Supplementary Materials

The supporting information can be downloaded at: https://www.mdpi.com/article/10.3390/ijms231810362/s1.
