# Supplementary material for: Cordycepin Inhibits Growth and Metastasis Formation of MDA-MB-231 Xenografts in Nude Mice by Modulating the Hedgehog Pathway
Source: Int J Mol Sci. 2022 Sep 8;23(18):10362. doi: 10.3390/ijms231810362 (PMC9499653; doi:10.3390/ijms231810362)
Supplement: Supplementary file 1 [file ijms-23-10362-s001.zip › ijms-1873212-supplementary.pdf]

Table S1: RNA sequencing data of 78 genes

Symbol	CON1 _count	CON2 _count	CON3 _count	COR1 _count	COR2_ count	COR3_ count	log2(FC)	Pvalue
SULF2	0	40	0	0	0	0	-7.29155	0.003837
SERPINA1	122	809	937	93	127	33	-3.20496	1.36E-09
CLIC5	1	30	1	1	0	0	-6.94642	0.004787
TBX18	0	19	4	0	0	0	-5.90689	0.002454
THNSL2	0	20	2	0	1	0	-5.49185	0.015665
VAV1	10	31	6	1	1	0	-5.47573	1.24E-05
AUTS2	1	5	47	3	0	0	-5.12928	0.001487
PTCHD4	1.06	4.14	1	0	0	0	-5.05889	0.04604
LPL	2	7	3	1	0	0	-4.80735	0.016554
MAOB	33	393	81	8	20	9	-4.04669	4.81E-07
IQGAP2	1	45	4	0	3	0	-4	0.002224
PAX5	9	8	2	0	2	1	-3.90689	0.013447
IGFBP5	33	821	90	34	24	24	-3.79941	8.57E-06
KRT5	2	140	2	0	11	0	-3.56071	0.009908
MUC5AC	16	117	38	7	10	2	-3.55459	5.17E-06
NKD1	90	1251	149	24	69	65	-3.38543	2.97E-05
STRA6	31	283	54	9	22	9	-3.34013	7.75E-06
BGLAP	5.52	14.45	13.13	2.35	1.12	1.07	-3.23133	0.000472
ALOX5	9	86	20	5	3	6	-3.20945	0.000238
VASH2	6	30	4	5	0	1	-3	0.002802
GNG2	34	241	35	13	6	22	-2.98674	0.000268
CP	9.02	24	20.02	8	2	1	-2.95777	0.000308
IGFBP2	1	20	8	0	2	2	-2.89162	0.012042
PROX1	15	52	12	9	1	12	-2.63227	0.023583
MMP7	18	100	31	15	2	13	-2.55149	0.001602
NKX2-1	8	0	13	2	2	0	-2.49185	0.031804
SHH	15	72	14	11	10	5	-2.4021	0.002113
CD24	243	1155	292	120	192	104	-2.37254	2.81E-06
GLI2	36	64	26	30	35	13	-2.34852	0.037917
PDE3A	6	55	14	18	3	4	-2.12553	0.018462
COBL	21	68	15	13	5	16	-2.11189	0.016826
SALL2	5	42	31	2	13	7	-2.07923	0.020785
SOX2	7	38	14	11	1	6	-2.06529	0.015957
WNT5A	5	19	48	6	2	2	-2.05889	0.00028
SFMBT2	28	23	188	5	44	16	-2.01057	0.01743
LGR5	5	30	9	0	9	2	-2	0.025145
ID3	131	503	469	117	85	121	-1.96963	7.10E-06
SCIN	8	46	51	5	23	3	-1.88085	0.010495

HCLS1	12	62	48	9	12	15	-1.85609	0.003886
DZIP1	38	76	23	12	18	17	-1.82823	0.003979
LEFTY1	1.32	27	9	5	6	0	-1.78727	0.033022
TNFRSF1B	5	26	25	9	8	7	-1.75899	0.034105
CYFIP2	21	98	30	13	23	18	-1.65849	0.010381
SERPINF1	404	2359	1832	1133	383	397	-1.62408	0.000114
HEY1	48	117	77	48	32	23	-1.60145	0.000482
TUBB2B	20.73	68.01	43.67	12.68	22.21	18.13	-1.54889	0.00867
AXIN2	105	414	179	93	185	65	-1.54224	0.004865
ID4	9	18	18	6	13	2	-1.53916	0.035049
SSTR2	19	90	34	25	29	11	-1.53766	0.012809
TSPAN8	40	95	56	30	58	6	-1.50627	0.015757
MASP1	1	10	3	0	0	1	-1.5025	0.015952
SIX3	7	29	23	12	4	9	-1.47249	0.033136
GDF5	6	18	11.27	3	5	6	-1.46949	0.044061
SDC2	163	482	335	115	202	79	-1.45741	4.31E-05
BOC	37	243	148	97	50	29	-1.45337	0.002069
CDH11	1708	3705	1344	670	1976	644	-1.43959	0.000756
TGFB2	320	1055	278	208	493	128	-1.4046	0.004679
INHBB	69	149	79	74	77	20	-1.28325	0.008301
CFH	293	942	273	188	585	89	-1.26103	0.023779
TIAM1	325	631	437	199	326	264	-1.25945	0.001749
PDGFRB	185	413	229	121	260	107	-1.23075	0.007054
KRT14	1528	1227	342	940	950	191	-1.23019	0.017447
CDON	327	891	370	173	370	225	-1.21666	0.001223
CLU	225	616	460	220	362	116	-1.15361	0.000518
MAOA	124	543	303	276	202	105	-1.13548	0.005627
PECAM1	110	324	205	103	142	111	-1.10912	0.007976
CA12	225	732	934	520	271	299	-1.10449	0.002211
SDR16C5	64	162	82	77	88	29	-1.09507	0.018418
DDR1	1690	3296	4819	3074	2505	1296	-1.03752	0.000137
NEO1	115	505	341	158	181	203	-1.02829	0.021055
SPARC	102	446	1047	132	68	289	-1.02742	0.01096
ID1	755	2652	2063	881	933	1230	-1.02728	0.004988
GNAO1	25	106	114	20	36	32	-1.02466	0.005073
KLF9	130	394	248	195	179	114	-1.01004	0.006491
SCO2	290.88	344.94	473.02	767.1	505.51	302.98	1.057378	0.026082
CCL2	9	14	15	82	26	17	1.198381	0.048085
MMP2	279	335	228	578	2714	91	1.53791	0.031383
FLT1	0	2	3	21	11	2	3.807355	0.021803
